# What does family involvement in care provision look like across hospital settings in Bangladesh, Indonesia, and South Korea?

**DOI:** 10.1186/s12913-022-08278-7

**Published:** 2022-07-16

**Authors:** J. Y. Park, J. F. Pardosi, M. S. Islam, T. Respati, K. Chowdhury, H. Seale

**Affiliations:** 1 School of Population Health, Faculty of Medicine, University of New South Wales, Sydney, NSW Australia; 2 School of Public Health & Social Work, Queensland University of Technology, Brisbane, QLD Australia; 3 International Centre for Diarrhoeal Disease Research, Bangladesh (icddr,b), Dhaka, Bangladesh; 4 Faculty of Medicine, Universitas Islam Bandung, Bandung, Indonesia

**Keywords:** Healthcare-associated infections, Hospital infections, Family caregiver, Infection prevention and control, Patient care, Caring culture, Private caregivers, Family involvement

## Abstract

**Background:**

Family members provide care whilst staying in the patient’s room across a range of cultural settings, irrespective of resource availability in many Asian countries. This has been reported as a contributing factor to the spread of several outbreaks, including COVID-19. Despite these reports, very little is known about the risk of healthcare-associated infection (HAI) transmission related to the involvement of family and private carers in the clinical setting. As a starting point to understanding this issue, this study aimed to provide insights regarding the patient care activities undertaken by family and private carers and the guidance provided to these carers around infection control measures in hospitals located in Bangladesh, Indonesia, and South Korea.

**Method:**

A qualitative study involving 57 semi-structured interviews was undertaken in five tertiary level hospitals across the selected countries. Two groups of individuals were interviewed: (1) patients and their family carers and private carers; and (2) healthcare workers, including doctors, nurses, hospital managers and staff members. Drawing upon the principles of grounded theory, an inductive approach to data analysis using thematic analysis was adopted.

**Results:**

Five main themes were generated from the analysis of the data: (1) expectation of family carers staying with a patient; (2) residing in the patient’s environment: (3) caring activities undertaken by family carers; (4) supporting and educating family carers and (5) communication around healthcare-associated infection and infection prevention and control.

**Conclusion:**

Based on the types of activities being undertaken, coupled with the length of time family and private carers are residing within the clinical setting, coupled with an apparent lack of guidance being given around IPC, more needs to be done to ensure that these carers are not being inadvertently exposed to HAI’s or other occupational risks.

**Supplementary Information:**

The online version contains supplementary material available at 10.1186/s12913-022-08278-7.

## Background

While most SARS-CoV-2 (COVID-19) cases have been community-acquired, there has been a smaller proportion associated with healthcare settings. Researchers from the UK reported that 1 in 10 cases of COVID-19 was healthcare-associated infection (HAI) [1], whilst a Chinese study suggested an even higher proportion at 41% of cases [2]. Early in the pandemic (March 2020), the incidence of patients with COVID-19 exceeded the availability of single isolation rooms in many UK hospitals [3]. Studies exploring these HAI COVID cases have focused on the role of the healthcare worker (HCW) [4, 5], but in some situations, the source of the infection has been linked back to the patients’ family members and/or privately hired carers [6, 7]. Previously the role of family carers in the clinical setting was linked to the spread of Middle East Respiratory Syndrome (MERS) during an outbreak in 2015 [7–10]. A recent COVID-19 outbreak report noted the role of multiple occupancy room settings, where patients’ caregivers stay next to the patient, as potentially facilitating the spread of the virus [6, 7, 9]. Despite these studies and others [11–13] acknowledging the role of family carers in the transmission of infections during outbreaks, little attention has been given to this issue.

The involvement of family members in the care of patients is commonplace in many Asian countries. Family carers and/privately hired carers are not considered HCWs but may provide continuous care. The types of care provided can include: (1) direct contact activities (i.e. changing the position of the patient, toileting, sponging, assisting with ambulation); (2) indirect contact (i.e. administering medication, making beds), and (3) aerosol-generating procedures (i.e. feeding via NG tube, and suctioning) to their sick family members at the bedside [14–21]. Even during the COVID-19 pandemic, when bans have been placed on visitors attending family members or friends in hospitals, reports have circulated that family members have been able to continue to provide care onsite [8, 10, 22].

While the WHO’s COVID-19 infection, prevention and control (IPC) guideline [23], includes a statement regarding the management of visitors (defined as ‘*Individuals accessing the healthcare facilities not to directly seek healthcare service, but to physically be present with a patient. Visitors provide various levels of support to patients during treatment (personal, social, psychological, emotional, and physical).*’), the sentiment focuses on family members or friends being onsite for compassionate end of life care visits. The guidelines include no real acknowledgement of visitors providing physical or care support.

A lack of recognition regarding the degree to which family members are involved and the types of activities being undertaken may contribute to why this risk group has not been acknowledged in the international infection prevention and control guidelines [23, 24]. To support future updates of IPC guidelines, this study aimed to provide a rich understanding of the context in which direct patient care activities are provided by family carers/private carers, the types of activities and the guidance provided to carers regarding the IPC measures to protect their sick family members and themselves. This study focused on three Asian countries, namely Bangladesh and Indonesia (reflecting examples of low-and-middle income settings) and South Korea (reflecting a high resource setting).

## Methods

### Study design

This research used a qualitative approach to understand the phenomenon of care provision by family/private carers in a context significantly influenced by cultural values and social norms. It also explored the level of engagement in IPC measures among patients and their family carers. Using a social constructionism paradigm [25, 26], the meaning of ‘care provision’ was understood as a socially constructed reality. Care provision is an embedded practice which can be best understood through a social constructionism paradigm. The outcomes presented in this study are part of a larger body of research being undertaken focused on examining whether the care provided by family carers constitutes an infection risk. As a starting point, we felt that it was critical to understand the current landscape of family carers’ involvement in the care across the focus settings. A semi-structured interview was selected as the most appropriate data collection approach. It provides the opportunity to capture a rich understanding of the practices and explore the different stakeholders’ thoughts, feelings, and beliefs towards the caring practices being used at the hospitals.

### Study setting

The data was collected from five tertiary level hospitals across Bangladesh, Indonesia, and South Korea between July 2019 to February 2020. Most of the data were collected prior to the emergence of COVID-19, with only two interviews undertaken during the pandemic. These countries were selected as they: (1) reflect examples of the spectrum of socio-economic countries in Asia, low-income countries (LIC), middle-income countries (MIC), and high-income countries (HIC); (2) are known to have a culture of family members providing care; and (3) had local researchers who could undertake the interviews in the language(s), and who understood and had access to the hospital settings. These country settings also provided an opportunity to explore the hypothesis that the involvement of the patient and their family/private carers in the phenomenon of care provision occurs across cultural settings, irrespective of resource availability. As noted by Creswell [27] and Trutty, Rothery and Grinnell [28], the capacity to be immersed in the natural setting is an imperative component of rigorous data collection. Participants were recruited from general wards hosting adult and lucid patients, excluding high-dependency wards, paediatric wards, psychiatric wards, psychogeriatric wards, and palliative care wards. Eligibility criteria for participants are listed in Table 1 below.

**Table 1:** Eligibility criteria for participants

Participant group	Requirements
**Patients:**	Aged 18 years or over Admitted into hospital for at least 24 hours Had a family member or private carer during the hospital stay
**Family members/Private carers:**	Aged 18 years or over Provided care for a patient for at least 5 hours in a hospital
**Healthcare workers:**	Had worked on the ward for at least one month Provided clinical care to patients on a ward.

### Recruitment

This study utilised a range of sampling approaches, including both convenience and purposeful, as well as snowball recruitment. Convenience sampling was first applied to identify study sites that could provide access to the different groups of participants [29]. Once the study sites were confirmed, purposeful sampling was applied to recruit the study participants. During the data collection, the existing participants were asked to alert other patients, family members and private carers in the same ward about the study. Thus the snowball technique occurred. The snowball method was also used to identify other HCWs for the interviews. Participant recruitment continued until the researchers felt there were no new ideas or new issues raised and felt the data saturation was reached.

### Data collection

Semi-structured interviews were undertaken with two different groups across the three countries: Group 1- Patients, family carers and private carers; and Group 2- healthcare workers, including nurses, doctors, hospital managers and staff members responsible for implementing infection control strategies. Interview guides developed separately for each group reflecting their roles and responsibility were translated (by native speakers into Bengali, Bahasa Indonesia and Korean) and pre-tested in Bangladesh and Korean sites prior to use. The local data collection teams reviewed feedbacks and comments received during the pre-test process, and the interview guides were amended accordingly. The interviews with the participants from group 1 were conducted either at the patient’s bedside or in an empty patient room available as per their preference and availability. Interviews with the participants in group 2 were done in various locations such as an empty staff room, a café in a lobby, a vacant manager’s office or a patient room depending on the availability. Some of the interviews were done in a group setting, i.e., a patient and their family carer or two healthcare workers together, often because of time constraints. All interviews were conducted in participants’ native languages, i.e., Bengali, Bahasa, and Korean, by researchers who were native speakers. Interviews were conducted by JYP for South Korea (KR) site, KC and his research team for Bangladesh (BD) site, and TR for Indonesia (INA) site. The duration of the in-depth interviews was 17 minutes to 55 minutes. All interviews were audio-recorded for the purpose of analysis with the agreement of all participants.

### Data management and analysis

The researcher who conducted the interviews transcribed the interviews verbatim in the local language, and the transcripts were then translated into English professionally for the Bangladesh and Indonesian sites. JYP, fluent in English and Korean, was the first author to translate the Korean interviews. The back-translation method was applied for the first two translated transcripts by an independent person who is qualified to interpret English and Korean to verify translation accuracy.

Rigour of the data was assured using Lincoln and Guba’s evaluative criteria [30]: credibility, transferability, dependability, and confirmability. For example, to ensure credibility in the data collection process, several strategies were applied. Firstly, data source triangulation was applied by collecting data from multiple information sources, i.e., patients, family carers, privately hired carers and HCWs, including nurses and doctors across three countries. Also, participants were selected from multiple sites; thus, site triangulation was achieved. In recruiting participants, purposive sampling supported diversity within a given population and included participants who have experience and insights into care provision in acute care settings. Next, as multiple researchers were involved in the study, researcher triangulation was achieved. Fourth, the research team had a series of training sessions, constant meetings throughout the data collection phase and reflection meetings during the analysis phase to share experiences. Thus, the interpretation of the data would be confirmed by the readers.

NVivo 12 was used for coding and data management. Drawing upon the principles of grounded theory, thematic analysis using an inductive approach was applied to identify the themes [31–33]. Braun & Clark [34] noted that thematic analysis is flexible to any theoretical framework and a ‘useful method for examining the perspectives of different research participants, highlighting similarities and differences, and generating unanticipated insights’, especially for a large data set. The interviews were repeatedly listened to, and notes were taken to ensure that all the relevant ideas were captured. An iterative process of data analysis was conducted. The initial phase involved familiarisation via multiple readings of the interview transcripts. The transcripts were coded initially, and a coding scheme was developed. The first author, JYP, was responsible for developing initial codes, then in consultation with HS and JFP, the final codes were developed. To ensure an accurate representation of the participants’ opinions and experiences, we attempted to use the participants’ voices to describe the codes’ naming and description.

## Results

Across the three countries, a total of 57 interviews with 64 participants, including 6 group interviews, were conducted. Of the 64 participants, 27 healthcare workers (21 female nurses and 6 male doctors), 16 patients (12 female and 4 males), 20 family carers (16 female and 4 males) and 1 privately hired female carer were recruited across the three selected countries as below (Table 2).

**Table 2:** Characteristics of interview participants across selected countries

Characteristics	Bangladesh	Indonesia	South Korea
Total (*N* = 64 participants)	20	19	25
**Group 1**	12	8	17
Patient	6	2	8
Family carer	6	6	8
Private carer	0	0	1
Gender			
Female	8	7	14
Male	4	1	3
Age			
≤ 25	2	3	1
26–35	7	2	5
36–45	3	2	0
46–55	0	0	3
56–65	0	0	8
> 65	0	1	0
**Group 2**	8	11	8
Doctor	4	2	0
Nurse	4	9	8
Gender			
Female	5	7	8
Male	3	4	0
Age			
≤ 25	1	0	1
26–35	4	6	6
36–45	3	3	1
46–55	0	2	0
56–65	0	0	0
> 65	0	0	0

**Fig. 1:**
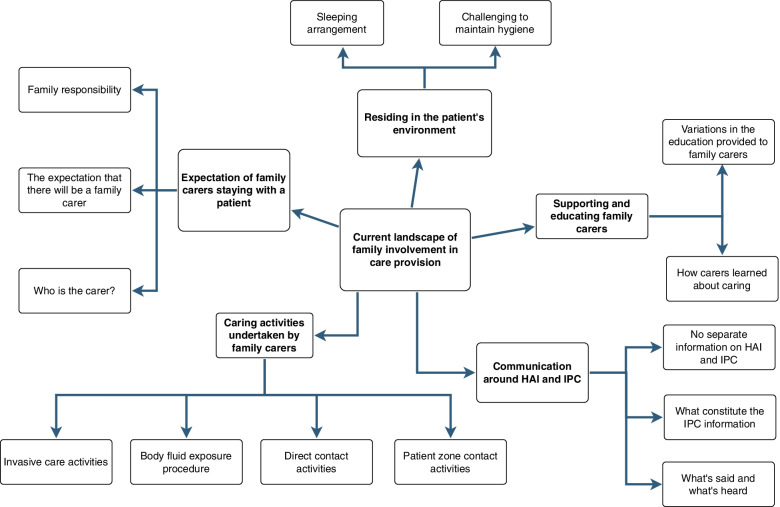
Themes in a mind map

The study findings showed 5 themes and 14 subthemes, as below (Table 3 and Fig. 1).

**Table 3:** Themes and subthemes from the qualitative findings

Theme, subthemes	Description
Theme 1	Expectation of family carers staying with a patient
Subthemes	▪ Family responsibility ▪ The expectations that there will be a family carer ▪ Who is the carer?
Theme 2	Residing in the patient’s environment
Subtheme	▪ Sleeping arrangement ▪ Challenges to maintain hygiene
Theme 3	Caring activities undertaken by family carers
Subtheme	▪ Invasive care activities ▪ Body fluid exposure activities ▪ Direct contact activities ▪ Patient zones contact activities
Theme 4	Supporting and educating family carers
Subtheme	▪ Variations in the ‘education’ provided to family carers ▪ How carers learned about caring
Theme 5	Communication around Healthcare-associated infections (HAI) and Infection prevention and control (IPC)
Subtheme	▪ No separate information on HAI and IPC ▪ What’s said and what’s heard ▪ What constitute the IPC information

### Expectation of family carers staying with a patient

#### Family responsibility

The expectation that a family member will stay with a patient during hospitalisation was expressed by participants in both groups across all country settings. What differed were the reasons provided for why the family carers should stay with a patient. In some instances, it was framed as a ‘given’ that a family member would accompany the patient: *“one person should stay right next to a patient (KR, Fc, 002*)”. Others framed it as a ‘family responsibility’, including a carer who was attending her husband in hospital in Indonesia: *“I take care of his medication…. sponge his body in the bed…and shower him…. Just in case…something might happen to him in the toilet. Keep my eyes on him, wipe him after he is done too… well, it is inevitable, since it is my job as a wife…. (INA, Fc,002)”*. Similar statements came from family carers in BD and KR: *“It is my responsibility to my mother (BD, Fc, 003)”* and *“I help my wife because we are family (KR, Fc, 004)”.*

#### The expectations that there will be a family carer

The expectation that a family carer will stay was significantly strong among HCWs, with participants using phrases such as “should have”, “has to”, and “must”. For example: “*all patients should have one family carer staying with patients in this ward (KR, Nr, 001)”*. When describing the expectation that a family member would stay with the patient, it was common to highlight that this also entailed the family member providing ‘care’.

The HCW spoke about the need for family carers to provide basic nursing care to the patients as a solution to the high nurse to patient ratios: “*Because we cannot possibly do the position change every 2 hours for them…. whilst caring for 15 patients per shift…. without a family carer or private carers’ help. (KR, Nr, 002)”.* Another nurse related the need for support from family carers because of her high workload*s: “If people (visitor) are strictly not allowed, then, the health facilitators (HCWs) would have to do these (activities of a carer). But it is not surely possible in Bangladeshi context”. (BD, Nr, 002)*. Family carers also commented on the staff shortages and the need for family carers to step in.

The expectation that there will be a family carer staying with a patient did not waiver even if the patient was placed under transmission-based precaution with MRSA or VRE. All participants agreed that a family carer would stay in the isolation room and continue to care for the patient without any exception. When asked if a family carer stays with a patient who was isolated with confirmed VRE, a healthcare worker responded, “*One carer has to stay with a patient. If there is no family carer, a private carer must stay (KR, Nr, 007)”.*

During the COVID-19 pandemic, the expectation of a family carer staying with a patient continued. When asked if a family carer is allowed despite the strong IPC measures introduced for COVID-19, a participant said: “*Yes, we advised that one person can stay*. *And, if a patient is at risk of fall, we strongly advise that a family carer must stay” (KR, Nr, 007).*

#### Who is the carer?

Interviews with family members revealed that the role of the ‘family carer’ is shared, with a range of ‘family’ members taking turns to care for the patient.

*My husband and daughter are looking after me from my family…. My sister…. sisters have come, sister’s son has also come. Like that. My sister-in-law is due to come after my...my daughter leaves. My sister-in-law will take care of me when she comes. (BD, Pt, 003)*

*I usually (husband of a patient) stayed here all day with her…then our son comes in the evening after work…other son who works away comes on his day off. But our daughter-in-law took a leave to stay with her for a week when she had surgery. She did it twice for her, and she took good care of her. (KR, Fc, 004)*

*My husband’s younger sibling usually replaces me while I am gone. (INA, Fc, 002)*

When a family member cannot fulfil the role, a private carer is arranged*: “We ask a family carer to come……... (if not available) ... We can use a free private carer…from the social work department (KR, Nr, 005)”.* According to one participant, who was a private carer hiring a private carer was made between a patient and a private carer. Although the list of private carers was provided to a patient by HCWs, the actual arrangement and hiring were privately made by patients or their family members. Interestingly, in an interview with a HCW, it was noted that getting a private carer was suggested to the family when they appeared to be incapable of carrying out the role: *“For those who are hard to learn or who are not interested in learning the skills, we let them know they can get a private carer.” (KR, Nr, 001).*

### Caring activities undertaken by family carers

Family carers spoke about undertaking a range of different care activities. These have been grouped into four categories based on the type of activities and whether it involves contact with their patient and/or their environment: 1) invasive care activities, 2) body fluid exposure procedure, 3) direct contact activities, 4) patient zone contact activities. Examples of quotes for each category are listed in Table 4 (see Supplementary files: Table 4).

### Residing in the patient’s environment

#### Sleeping arrangement

There were mixed responses from family carers across the three countries when asked where they slept whilst caring for their loved ones in a hospital. Participants from KR said they slept in a foldout bed between the patients’ beds, whereas family carers from BD and INA reported that they stayed on the floor. A participant from INA who had been caring for her husband with a heart condition said about her sleeping arrangement:

*I sleep on the floor. ...I brought my mat. It is cold, actually, and I obviously could not sleep on the bed, since it’s small… I sleep on the floor with the mat I brought (INA, Fc, 002).*

A family carer from BD who cared for her mother with a broken leg also has a similar sleeping arrangement: *“For myself, I clean this down place very well. I keep a mat on it. After that, I use a Katha or blanket on it. Then I clean myself very well, and I sleep there (BD, Fc, 003).”* A nurse from INA mentioned, “*They sleep on the floor. Actually, it is not allowed. But since they travel far from their hometown, sometimes we let them.” (INA, Nr, 006).*

#### Challenges to maintaining hygiene

Some HCWs spoke about the challenges of family carers maintaining their hygiene whilst staying at the hospital:

*They cannot maintain hygiene properly…. Before coming to the hospital, they were completely healthy, but after attending a patient, for example, for ten days, they also became infected. We only focus on the patient, but at the same time, his attendants also get infected. Most of them (patients and attendance) get a viral infection in the winter season. These infected attendants are serving the patients, and thus they are also getting infected (BD, Dr, 008).*

Several participants brought up their concerns about sharing a bathroom during the interview when asked about their stay in a patient room. Family carers from KR said that there was a separate toilet available for family carers when asked if they had to share a bathroom with other patients in a patient room.

*The patient is doing here…and family carers can use the toilet outside…But it is a bit…how to say...relating to infections... things like that. (KR, Fc, 003)*

### Supporting and educating family carers

#### Variations in the ‘education’ provided to family carers

There were mixed responses regarding the provision of education or guidance to family carers about providing care to patients. When asked if there was a standardised education around care activities for patients and their family carers that they had to provide to their patients, a nurse from KR stated: *“Yes, about basic stuff…we give them guidance. But some of them have a lot of experience in caring, so they do it without any training from us. Other than that…. If she is a first-time carer, we provide education about position change…and go through some tasks that they should be careful about (KR, Nr, 002)*. The education was only provided in situations where things went wrong or for the ‘first-time carer’*.* Another participant confirmed that they *don’t teach every single carer each time when they come* unless a patient is at risk of developing complications such as the development of bedsores or aspiration from L-tube feeding if care was compromised.

Because many family carers were not informed of how to take care of their loved ones, some reported experiencing difficulties when carrying out the activities. A family carer from INA who cared for her sister stated that “*she was a little baffled to change her sister’s clothes as a patient was attached to an IV drip on her hand”.* When asked if she received any information or education, she said *she had never been taught (INA, Fc, 005).*

Contrastingly, a few HCWs said they have provided information to family carers about caring activities and explained how to. A nurse from KR stated that family carers were continuously given education until they grasped the basic sense of how to carry out the caring activities:

*We are continuously educating them about how to do it until they get used to do...they are unfamiliar with those care activities because they are not healthcare workers. For those who are hard to learn or who are not interested in learning the skills, we let them know they can get a private carer. However, getting a private carer is quite expensive. Some of them cannot afford private carers, and we must repeatedly educate them. (KR, Nr, 001)*

However, a HCW from BD said that the education for family carers was no longer offered*: “We had a training team. It continued for about six months. Our two/three senior staff nurses took health class for one hour with the patient and their attendance each day of the week. They instruct them on what should be done for the sake of their patients. I do not know why they stopped the program (BD, Nr, 001).”*

#### How carers learned about caring

Observing how other people do things was how participants said how they learned about caring for a patient. Some said they watched how nurses looked after their patients: *I observe the process of how nurses look after my mother. For example, legs need to keep straight this way, and it is important to avoid bending the leg” (BD, Fc, 003).*

Others said they observed other family carers and learned from their practice*: “She is learning from observing the patient next to us. Like other daughters taking care of their mothers while maintaining the rules, my daughter is following the same steps they are doing. Only this way... my mother will get well. This way… she learns from observing others. She observed, how other daughters here are take care of their mothers for sixteen days. And how they take care of their mothers. She does it by observing this way. She is doing it within this norm. (BD, Pt, 003)”.*

Other than observation of others, learning from others was noted as a learning method for carers.

*Private carers are teaching each other…because many of them are there…. sometimes, there are 2 or 3 private carers in the room…so among themselves…and if one private carer is not available, the other private carer notifies us about another patient…they are teaching each other and helping each other in the room. (KR, Nr, 008)*

Some participants said they searched for information online and learned themselves.

*I searched on the internet about her surgery, recovery, any complications and so on...I searched those…but those on the internet are written by unknown individuals, and mom’s case is not the same as those on the internet…so things I couldn’t find on the internet or other symptoms that are not on the internet, but mom was suffering, I had to ask nurses…But mostly, basic things are there on the internet. (KR, Fc, 003)*

### Communication around HAI and IPC

#### No separate information on HAIs and IPC

HCWs recalled that they *provided information about hand hygiene when they explained the hospital stay* on patients’ admission:

*First, after a patient is admitted to the hospital, there is something called orientation. It includes an orientation to the room where they will be staying later. We cover the facilities that can be used and what they should do to prevent infection transmission such as washing hands. (INA, Nr, 007)*

However, patients could not remember getting any separate information around HAI, and IPC was not separately discussed or communicated to patients and/or their family carers. A patient’s response also suggested that the IPC information was embedded in the general information. In the interviews, HCWs said that they *do not explicitly talk about IPC measures because posters and pamphlets attached to the wall in the patient’s room, in the bathroom and everywhere. (INA, Nr, 005 & KR, Nr, 004)*. Very few of the patients’ interviews mentioned seeing posters about hand hygiene and PPE.

#### What’s said and what’s heard

There were contrasting statements regarding what was said and heard between HCWs and patients/family carers around IPC information. Several HCWs across three countries stated that the information about hand hygiene, including alcohol-based hand rub (ABHR), was given to patients and their carers during their stay.

*We tell patients’ families that they need to wash their hands every time they touch patients. If washing hand with water and soap is not convenient, we ask them to wash hands with an alcohol-based hand rub. We have alcohol-based hand rubs at each bed and the gate of the ward before the screen door. (KR, Nr, 001)*

*As we constantly educate the patients, we usually educate them as soon as they are admitted or hospitalised. We give them advice about hand hygiene. There are orders to do it, and patients are being taught how to wash their hands properly according to the orders along with the 5 moments. When they are required to wash hands, and how to do it. (INA, Nr, 011)*

However, most patients and their family carers shared different experiences regarding the communication around IPC information. When asked if any information about HAI and/or IPC measure were explained, a patient from BD stated:

*No, they did not give me any...They did not tell me about the ways like, ‘Follow this, you will not get infected.’ I did not get any guidelines. But I do not know if they have given anywhere else. (BD, Pt, 006)*

The statement from a family carer from INA below indicated that there was no communication around ABHR and hand hygiene.

*I think any information about how to take care of the patients properly. For example, they should tell us where we can get the hand sanitiser, and they should constantly remind us to use the hand sanitiser before we do anything…. Also, we should be prompted to be careful when there is a visitor. (INA, Fc, 005)*

Similarly, participants’ responses from KR pointed out that the information around ABHR was not conveyed to family carers.

*Actually, I did not know what it (ABHR) was until now. Also, we are allowed to use it…. Frankly speaking…nurses use it occasionally. I only knew now that we could actually use it. I have only seen that at the door of the room. (KR, Fc, 010)*

*Frankly speaking, I have not used it at all…. In the morning, the professor who did the surgery for my daughter came for the round. And I saw him using this as well as his fellow doctors. Then I knew what this thing was for. I started to use this since then. I learned by seeing it...........I wonder why he was doing like this (rubbing motion) …I thought this was a lotion. (Kr, Fc, 007)*

#### What constitutes IPC information

Although many participants said they received no information about HAI and IPC measures, a few patients and family carers said they did. When it was elaborated further about what information was discussed and delivered, several interesting things were noted about what was taken as IPC information by patients and family carers. The family carers spoke about the need to keep the patient ‘clean and hygienic’ but did not elaborate on the types of IPC activities that they should carry out.

*Yes, they (nurses of this hospital) have told us that we should stay clean and hygienic. If we stay clean and hygienic and keep ourselves aware of being neat and clean, there will be no problems to the stitches. We have been told… everyone has been told*. *(BD, Fc, 001)*

*Upon arriving at medical, I am learning this...The doctors and nurses...They said that a patient has to stay clean at all times in an operation hall. Otherwise, infections may occur. Because ultimately, the patient will suffer. (BD, Pt, 003)*

HCWs’ responses also indicated that only brief instruction was given to patients and family cares as IPC education.

*We cannot provide health education all the time*. *When the patient gets admitted*, *we tell them*. *Besides, when we give them medicines, we ask about their health* *and tell them to do things in certain* *ways*. *We tell them to wash their hands or not to worry*… *Aaa*… *I meant, whenever we go to see them, we tell them to stay neat and clean as much as possible*. *(BD, Nr, 005)*

*There is information about hospital stay… but we tend to read it through to them briefly. We mention hand hygiene like...it is good to do the hand hygiene…. but we don’t show them how to do it. (KR, Nr, 007)*

## Discussion

The five themes that emerged from the interviews undertaken across the three countries highlighted the current situation regarding family involvement in the care provision. The first theme, ‘*Expectation of family carer staying*’, revealed the reasons and/or factors associated with this care arrangement. Many family carers framed their involvement as a ‘*family responsibility’,* which is closely related to filial piety. Filial piety, or ‘*the obligation of family members to care for each other, especially for older generation*’ [35], has been highly valued in many Asian societies [35–37]. As Crotty [38] noted, the provision of care as an expression of filial piety is learned and consolidated as morally acceptable behaviour [35, 39–43]. During the interviews, the provision of this kind of care was described as a natural process and morally appropriate behaviour. This mutual expectation appears as if a tacit agreement was made without being officially documented in any policies or guidelines. The lack of reference in guidelines may also be linked to the fact that the provision of care by family members has been a longstanding custom across multiple Asian countries [17, 39, 43–45].

In comparison to many high resource countries, for instance, in the US, the nurse-to-patient ratio is often recorded at being1:4 and 1:6 depending on the acuity of patient care needs. Whereas ratios in many low resource countries can be much higher at 1:13 in Bangladesh, 1:9 in Indonesia, 1: 40–60 in the Philippines, and 1:30 in Vietnam [18, 46–48]. Aligned with the findings from previous studies [21, 40, 49–52], familial involvement in care provision appears to be a strategy to overcome this difficult situation, with family or private carers acting as an alternate workforce. Previously studies have documented that approximately 90% of patients have a caregiver during their admission, and of the patients with caregivers, 25% had paid a private caregiver in Korea [40, 53].

The second identified theme was ‘*Residing in the patients’ environment*’. It has been previously acknowledged [54] that high numbers of people and increased activities in the patient care environment can significantly contribute to the contamination of surfaces with microorganisms and the increased frequencies of hand contact with surfaces. While this study did not quantify the number of patients and family members in each setting, from the interviews, there was a clear sense that the family carers are present in these settings for long periods of time, both day and night. The high number of people in these settings (and the subsequent contact with the patient and their environments) may result in increased transmission risks of microorganisms from the environment to patients, family carers, and healthcare workers. A recent study from Bangladesh [55] examined the association between the hospital environment and the transmission of respiratory infections among patients, family carers and HCWs. They found that the overcrowding conditions in the hospitals due to the large numbers of family carers staying in between individual patient beds, on the beds, and under the beds was contributing to the transmission. This issue has also been reported during a range of pandemics and outbreaks, including COVID-19 [7, 8], MERS [56], Nipah [57] and Avian influenza A(H5N1) [58]. One of the key contributors to transmission in these settings is the impact of overcrowding and the inadequate spacing between patient beds on the capacity to complete the appropriate hand hygiene moments [59].

The third theme focused on the *caring activities* that family carers provide to their sick patients. Findings from our study add to the body of evidence that the caring activities performed by family carers in Asian hospitals are different to those in Western countries [16, 17, 21, 43, 60]. While emotional support accounts for most familial involvement in Western countries, more physical care activities are commonly done by family carers in Asian countries. Given the types of procedures family carers reported doing, including *intravenous injections, nasogastric tube feeding, suctioning, wound dressing, and body fluid exposure activities*, the risk for HAI transmission from patients to their family members is heightened [23]. Despite this, the support and education provided to these carers varied across the settings. In many situations, the carers obtained their knowledge through informal routes such as observation, online searches, or learning from their family members. Given the potential for inappropriate and risky behaviours to perpetuate if carers rely on these informal routes, there is a need to identify a simple and cost-negative approach to support family carers to adhere to safe practices while providing care. Any approach could not place a burden on the existing staff members but instead would need to be tied into system and communication changes in the clinical settings.

The last theme that emerged from the study, *communication around HAI and IPC*, clearly highlights key gaps in communication between patients, family carers and HCWs. Meeting the patients and their family carers’ information needs is a crucial element in actively encouraging patients and their carers to undertake or adhere to recommended IPC measures [61–63]. Previous studies examining the importance of providing IPC information also demonstrated that patients’ compliance with IPC measures is affected by their understanding of the risk [64–68]. Sharing information related to the delivery of care and precautionary measures to reduce the risk of infection transmission could bring about good health outcomes for patients, family carers, and the community.

There were some limitations in the study. Although the study included multiple informants, i.e., patients, family carers, privately hired carers, and HCWs, we could only recruit one private carer. This was due to the unavailability of private carers during the data collection period and resource constraints of the study. Having only one private carer participant from one study site, i.e., Korea, may not reflect the perspectives of private carers adequately and may affect the internal validity of the study. Secondly, there was no doctor included from Korea. Thirdly, although we have collected data from three countries across various socio-economic settings, these findings may not represent or be generalised to other countries. Next, as addressed in the methods, some interviews were done in a group setting due to time pressure and feasibility. However, while there are advantages associated with group interviews, such as efficiency, we are also aware of the pitfalls. As Frey & Fontana [69] suggested, participants’ responses may not be as diverse as they could be.

## Conclusion

While WHO guidelines briefly address the caregivers’ involvement in the care of patients within IPC training guidelines, it is not clear whether these caring arrangements are adequately considered. Based on the types of activities being undertaken, coupled with the length of time family and private carers are residing within the clinical setting, coupled with an apparent lack of guidance being given around IPC, more needs to be done to ensure that these carers are not being inadvertently exposed to HAI’s or other occupational risks.

## Supplementary Information

**Additional file 1:** **Table 4.** Example quotes for each category of Caring activities undertaken by family carers.

**Additional file 2.** CORE-Q Consolidated Criteria for Reporting Qualitative Research.

## Data Availability

The datasets analysed during the current study are not publicly available due to participant confidentiality but are available from the corresponding author on reasonable request.

## Abbreviations

COVID-19: SARS-CoV-2, Coronavirus disease 2019
IPC: Infection prevention and control
HAI: Healthcare-associated infection
KR: South Korea
BD: Bangladesh
INA: Indonesia
Nr: Nurse
Fc: Family carer
HCW: Healthcare worker
Pc: Private carer
Pt: Patient
ABHR: Alcohol-based hand rub
MERS: Middle East Respiratory Syndrome
