# Supplementary material for: What does family involvement in care provision look like across hospital settings in Bangladesh, Indonesia, and South Korea?
Source: BMC Health Serv Res. 2022 Jul 16;22:922. doi: 10.1186/s12913-022-08278-7 (PMC9286761; doi:10.1186/s12913-022-08278-7)
Supplement: Supplementary file 1 — Additional file 1: Table 4. Example quotes for each category of Caring activities undertaken by family carers. [file 12913_2022_8278_MOESM1_ESM.docx]

Table 4. Example quotes for each category of caring activities undertaken by family carers

| Categories of care activities | Definition | Examples of quotes |
| --- | --- | --- |
| Invasive care activities | Care activities include below:   - Insertion of a needle into a patient’ skin including *subcutaneous injection* - Preparation and administration of any medication given via an invasive medical device such as *intravenous flush and nasogastric tube feeds.* - Administration of medications where there is *direct contact with mucous membranes* such as eye drop instillation, suppository insertion. - Disruption to the circuit of an invasive medical device such as *suctioning* of airways, *urinary catheter,* wound drains - Any assessment, treatment, and patient care where contact is made with non-intact skin or mucous membrane such as *wound dressing* | “I had to do everything... for say, doing patient's tests, maintaining his diet, everything. My father needed oxygen for 24 hours. In that case, I had to maintain everything. He had asthma. So, I also had to *give nebulization hourly, swabbing his body, giving insulin as he had diabetics*, taking care about his diet such as which food he should eat or which one to avoid; I had to do all of these.” (BD, Nr, 005)  “My father-in-law is taking an anti-biotic saline. I *pushed the saline myself into him*. Sister showed me how to open it and how to set it.” (BD, Fc, 006)  “For my mother, I help her to lean on her sides.... and I usually talk to her and try to communicate with her as well.*.. she uses NGT (nasogastric tube) so, I help her to eat.” (*INA, Fc, 006)  “Basically, they do most of basic care to patients…...... First of all….as mentioned on the consent form, helping patients with toileting including using bedpan, basic hygiene care and... if patients are really sick…. or when we are really busy, *family members are helping us with suctioning and L-tube feeding*… and suctioning and changing positions for patients are mostly done by family members.” (KR, Nr, 001)  “I help her get up, clean her clothes. The hospital is not very clean, so I send the clothes home to be washed…. I have to keep her clean so that she is free of germs, then I also have to check the wound from time to time to make sure it is clean*. The doctor also said to open the bandages sometimes so that it dries up, so I am also doing that*.” (BD, Fc, 004)  “Many patients are supposed to get food with an NG tube but not everyone can provide it. What would we do in that case? *We teach the service provider, how to feed with the NG tube. They are providing this favour*. They are cleaning everything and to some extent, they are also monitoring the patients as well.” (BD, Dr, 008) |
| Body fluid exposure activities | Care activities involves any potential body fluid exposure including:   - Contact with a used urinary bottle/bedpan, contact with sputum either directly or indirectly via a cup/tissue, cleaning spills of urine, faeces or vomit, after touching the outside of a drain, contact with used specimen jars/pathology samples - Contact with body fluid such as blood, saliva, mucous, urine, faeces, vomitus, | “Those patients who have a family carer or a private carer staying with them are generally requiring assistance with activities of daily living…...such as *changing incontinent pad,* running errands, helping with walking, or helping them getting into the wheelchair…. or changing positions especially for those who have bedsores.” (KR, Nr, 002)  “(when asked if FC helped more after surgery). on the next day of surgery, you know, *she was coughing up phlegm, so I got that on the tissue and threw that away*…that kind of thing...the nurses told us that they had to check the urine, so we called them when the urine bag was full. But emptying the bag was…the nurses took some for the test…*and we emptied the bag and threw that way*.” (KR, Fc, 010)  “They normally help patient with feeding, or when the patient doesn’t want to go to the bathroom or they can’t, then *the carer helps the patient with a bedpan*- assumed they are already being taught how to use it. That is all, I think, and to help with a shower.” (INA, Nr, 006)  “I *change her incontinent pads* and I tell nurses when there is a leak on her IV drip. Also, I feed her too. I take care of her medications and her drink as well. Sometimes she wanted to eat food from the outside. I would ask the nurses if that were allowed it or not.” (INA, Fc, 003)  “He (patient, father-in-law) tells us to massage all the body with wet cloth at noon. Then I(daughter-in-law) do it. I pour water on his head and help him using toilet*. He passes out urine by sitting.* He can’t move, so we do that way*. There is a bottle, urine bottle, he uses it by sitting*.” (BD, Fc, 006)  “When pain was really bad…. what was it?? The injection for pain. Nurse gave me the injection, but it did not help much so she gave me extra painkillers. *Then it made me vomit*…...When too much pain killer got in, that made me vomit. ……...Those things nurses did for me…. with my daughter. *But my daughter called nurses and cleaned up the vomit*.” (KR, Pt,003)  “I *clean her stool and urine from her bed.* I help in her feeding, I care for when and which is needed by her, I also provide medicine with the help of sisters*…. I clean her leg, stool, and urine.* I provide those duties and nursing which ensure her good health...I clean her stool and urine; I also help her using toilet. I also help her taking water and medicine. I feed her rice. I clean her body to protect her from nasty germs which spread.” (BD, Fc, 003)  “We have caregivers who feed the patient regularly and clean his urine and defecation.” (BD, Dr, 008) |
| Direct physical contact | - Caring activities associating touching a patient including assisting a patient to move, touching any medical device connected to the patient, - Any personal care activities such as *bathing, dressing, brushing hair* - Any non-invasive care activities such as taking a pulse/blood pressure, applying an oxygen mask, administering nebulised medication. | “If a patient is bedridden…or if a patient is not able to move after a surgery, family carers or private carers let us know about patients’ complaints, or they are actually taking care of a patient. Most of private carers are caring for cancer patients, bedridden patients, or neurology patients…. *they are helping patients with position change, or…those basic things*…things that are generally done by private carers.” (KR, Nr, 003)  “I had to do everything... for say, doing patient's tests, maintaining his diet, everything. My father needed oxygen for 24 hours. In that case, I had to maintain everything. He had asthma. So, I also had to *give nebulization hourly, swabbing his body……”* (BD, Nr, 005)  “They (family carers) came to physical contact. Because they feed him, brush his teeth, clean his body, change his dress, and take him to the toilet. *They do all these with physical contact.* So, there is a big chance of spreading infection.” (BD, Nr, 003)  “Basically, helping with meals…. *personal hygiene care and prevention of pressure sores*…*those activities require physical contacts with patients*…are done by the private carers.” (KR, Nr, 007)  “I *help her to go to the bathroom. Help her with her IV drip*... and such things. Also, when she wanted to *get up or get off from her bed*, *I let her lean on me*. And, when she eats her meal, take her meal. Help her *to get changed*, I guess…. And rub her to wash her.” (INA, Fc, 005)  “My aunt cannot walk. I help her to cover with cloths and *I help her to wear dresses*. I *massage her with oil*. I water her head. I help her to take medicine and I feed her. I *help her to sit and lifting*. I do all these. If she were at home, everyone could do. I have to do it all alone.” (BD, Fc, 002)  “For patients in third class, they *help patients with bath and toileting* like when they need to relieve themselves. For patients who has strokes, we will usually help them with the bathing for the first time. Then, they will help patients from the next bath.” (INA, Nr, 003)  “(when asked what activities family carers provide to patients) *changing bed linens or getting a patient gown*……or just helping patients with feeding or *helping them with walking around* the ward.” (KR, Nr, 004)  “Their services depend on the condition of the patients. They have to serve more to the patients who are senseless or unconscious. She sweeps the saliva which comes out from the mouth of the patient by sitting there. *Sometimes they need to hold many restless patients*....to help in taking medicine, feeding or such things, attendants do over more than ninety percent of these kinds of care.” (BD, Dr, 007)  “It is not hurting…Because I have been lying on the bed…yesterday, it was only my thighs got rubbed against each other…today even my knees are rubbed*…. Massage around legs makes the swelling better so…. I got my legs* massaged.” (KR, Pt, 005)  “Take care of his medication…. *sponge his body in the bed…and shower him*…. Just in case…something might happen to him in the toilet. Keep my eyes on him, *wipe him after he is done too*… well, it is inevitable, since it is my job as a wife…. I also feed him.” (INA, Fc, 002) |
| Patient zone | Touching the patient’s immediate surroundings when the patient has not been touched. Patient surroundings are bed, bedrails, linen, table, bedside locker, call-bell, personal belongings, chair, curtain around patient bed. | “…helping her with meals, *taking the meal tray away*…. other than that, nurses did all the other stuff…and letting the nurse know when the antibiotics or potassium finished. And like just now, *getting a patient gown for her*...and helping her go to the bathroom…just those.” (KR, Fc, 010)  “I think they mainly *help patients with meals.* The carers *also take care of the patient’s medications.* It is actually the nurses’ duty but sometimes the carers will help as well.” (INA, Nr, 002)  “We *took her by trolley (Wheelchair).* We rented a trolley to take her for X ray. We also used it for ECG and blood test. We did all the tests by using this inside and outside hospital.” (BD, Fc, 003)  “I *clean her bed,* or I clean her body, I *wash and clean around her*. I *clean her cloth*s to avoid dirtiness. I am taking care of her by doing all these.” (BD, Fc, 003)  “The bedsheet I have got from here. *She washes that every day. And she brings new bedsheets from home*…She has *brought a blanket, a pillow and all that.* She *washes all the necessary things with a soap*. She does it ...And she tries to keep me clean. She feeds me with her hands. (No utensils) ...She *checks if the medicines are timely taken or not* and make me take those. She checks on me if I have any problems. She discusses with the nurses if there is any problem. If there is anything to clean, she tells the cleaners... She helps me giving the toothpaste, the brush in the morning when getting fresh. She wipes my whole body with a towel as much as she can” (BD, Pt, 006)  “I help her get up, *clean her clothes. The hospital is not very clean, so I send the clothes home to be washed*…. I have to keep her clean so that she is free of germs, then I also have to check the wound from time to time to make sure it is clean. The doctor also said to open the bandages sometimes so that it dries up, so I am also doing that.” (BD, Fc, 004)  “…Besides, they need to bring medicine. *Some medicines are not available in the medical. They bring it from outside*.” (BD, Nr, 001)  “Feed the patient, change his cloth, take him to the toilet, *bring him medicine from outside, if needed bring him to the investigation or bring a sample, collecting reports.*” (BD, Dr, 003)  “In most of the cases, woman (family carer) stays, and a man (family carer) who stays brings medicines. Having two people staying over is tolerable nonetheless, but it has been seen that there are four/five people with one patient, then unthinkable trouble occurs.” (BD, Dr, 006) |
