# Supplementary material for: What does family involvement in care provision look like across hospital settings in Bangladesh, Indonesia, and South Korea?
Source: BMC Health Serv Res. 2022 Jul 16;22:922. doi: 10.1186/s12913-022-08278-7 (PMC9286761; doi:10.1186/s12913-022-08278-7)
Supplement: Supplementary file 2 — Additional file 2. CORE-Q Consolidated Criteria for Reporting Qualitative Research. [file 12913_2022_8278_MOESM2_ESM.docx]

**CORE-Q Consolidated Criteria for Reporting Qualitative Research**

Based on Tong, A., Sainsbury, P., & Craig, J. (2007). Consolidated Criteria for Reporting Qualitative Research (COREQ): A 32-item checklist for interviews and focus groups. *International Journal for Quality in Health Care*, *19*(6), 349–357.

| **No** | **Item** | | **Description** |
| --- | --- | --- | --- |
| **Domain 1: Research Team and Reflexivity** | | | |
|  | **Personal Characteristics** | | |
| 1 | Interviewer | JYP, KC, ZS, LK, TR | |
| 2 | Credentials | The three interviewers between them hold Bachelor, Master and PhD qualifications in Nursing science, Dental surgery, Social science in Anthropology, Epidemiology, and Public health | |
| 3 | Occupation | PhD candidate (JYP), Academic staff (TR) and assistant scientist (KC, ZS, LK) | |
| 4 | Gender identity | Female (JYP, TR, LK), male (KC, ZS) | |
| 5 | Experience and Training | Experienced researchers and interviewers have undertaken several qualitative studies and trained in interviewing and qualitative analysis. JYP is a novice in qualitative interviews but trained in qualitative research and interviewing. | |
|  | **Relationship with participants** | | |
| 6 | Relationship established | None of the interviewees was known to the interviewers before the interviews. | |
| 7 | Participants knowledge of the interviewer | The participants in this study were patients, family carers and healthcare workers from study sites across Bangladesh, Indonesia, and South Korea. The interviewers were not known to participants before the interviews were conducted. | |
| 8 | Interviewer characteristics | Established researchers with experience in social science and health services research, emerging infections. Collectively the interviewers have more than 20 years of research experience. | |
| **Domain 2: Study Design** | | | |
|  | **Theoretical Framework** | | |
| 9 | Methodological orientation and theory | Exploratory, social constructivism framework. | |
|  | **Participant Selection** | | |
| 10 | Sampling | Convenient, purposive, and snowballing, with participants sought across hospital settings from three study countries. Participants were also asked to identify participants to be interviewed. | |
| 11 | Method of approach | Walk around the ward and explain with participant information and consent forms. Existing participants to alert others. | |
| 12 | Sample size | 30-40 | |
| 13 | Non-participation | NA | |
|  | **Setting** | | |
| 14 | Setting of data collection | Conducted via a face-to-face interview with an audio recording device | |
| 15 | Presence of non-participants | Non-participants were present for some interviews when the interviews were conducted at the patient’s bedside in a multi-patient room or public place such as a cafe | |
| 16 | Description of sample | Participants were patients, family carers, private carers and healthcare workers from the tertiary level hospitals across Bangladesh, Indonesia, and South Korea. | |
|  | **Data Collection** | | |
| 17 | Interview guide | Provided in advance upon request | |
| 18 | Repeat interviews | No | |
| 19 | Audio/visual recording | Audio recording | |
| 20 | Field notes | Limited reflective notes were captured following the interviews. These notes were not included in the qualitative analysis. | |
| 21 | Duration | Ranged between 17 minutes and 55 minutes | |
| 22 | Data saturation | Purposeful sampling to ensure a range of experiences were captured across participant groups and study sites. As such, sampling continued until researchers felt no new ideas were raised. | |
| 23 | Transcripts returned | No | |
| **Domain 3: Analysis and Findings** | | | |
|  | **Data Analysis** | | |
| 24 | Number of data coders | JYP did the initial coding. HS and JFP contributed to the development and refining themes identified. All authors discussed codes and higher order themes. | |
| 25 | Description of the coding tree | No, not necessary, given the focus of the analysis is mainly descriptive rather than theory driven. | |
| 26 | Derivation of themes | Codes and concepts were derived through open coding of interview transcripts. These were then tested and consolidated into higher order themes. | |
| 27 | Software | NVivo 12 | |
| 28 | Participant checking | No | |
|  | **Reporting** | | |
| 29 | Quotations presented | Yes, selectively to illustrate findings | |
| 30 | Data and findings consistent | Yes | |
| 31 | Clarity of major themes | Yes | |
| 32 | Clarity of minor themes | No, the focus of this paper is on major analytic findings and higher-order themes, given the focus of the study. | |
